# Migration of Fish Bones to the Bile Duct Following Hepaticojejunostomy

**DOI:** 10.1002/deo2.70293

**Published:** 2026-01-28

**Authors:** Kenta Yoshida, Tsuyoshi Hayashi, Kazuki Hama, Ryo Ando, Haruka Toyonaga, Tatsuya Ishii, Toshifumi Kin, Masayo Motoya, Kuniyuki Takahashi, Yuko Omori, Satoshi Ota, Akio Katanuma

**Affiliations:** ^1^ Center For Gastroenterology Teine Keijinkai Hospital Hokkaido Japan; ^2^ Department of Pathology Teine Keijinkai Hospital Hokkaido Japan

**Keywords:** balloon enteroscopy, endoscopic retrograde cholangiopancreatography, fish bone, foreign‐body migration, pancreaticoduodenectomy

## Abstract

**Objectives:**

Migration of fish bones into the bile duct is rare, but can cause bile duct stone formation or acute cholangitis. Herein, we examine the characteristics of this phenomenon.

**Methods:**

This single‐center, retrospective study enrolled patients with a history of fish bone extraction from the bile duct by an endoscopic procedure at our hospital between June 2016 and November 2023. Patient characteristics, treatment details, and clinical course were assessed from the electronic medical records.

**Results:**

A total of 11 patients were enrolled, including 10 who underwent subtotal stomach‐preserving pancreaticoduodenectomy with Child's reconstruction and one who underwent extrahepatic bile duct resection with choledochojejunostomy. The median time between surgery and endoscopic treatment was 84 months (range 12–124). On computed tomography (CT), all fish bones were detected as high‐density dots or linear substances in the bile duct. Endoscopic procedures were performed using single‐balloon enteroscopy. All fish bones and coexisting biliary stones were successfully removed, with a median duration of 40.5 (range 9–54) minutes. Three cases of mild cholangitis were observed and resolved with conservative treatment. Two patients had recurrence, and all cases were successfully treated endoscopically.

**Conclusions:**

This migration may occur in patients with surgically altered biliary anatomy. Most migrated fish bones can be safely detected by CT, and treated by single‐balloon enteroscopy.

## 1 Introduction

Biliary infections, including cholangitis, cholecystitis, and hepatic abscesses, are primarily caused by biliary obstruction due to biliary stones or malignant diseases. Standardized methods for the diagnosis and treatment of biliary infections have already been established based on studies including a large number of patients, and have been described in several guidelines [1, 2]. Biliary foreign bodies can cause biliary infections, although prior reports are largely limited to case reports/series [3, 4]. Most biliary foreign bodies are remnants of medical material following biliary surgery or endoscopic retrograde cholangiopancreatography (ERCP) procedures, whereas a smaller proportion result from bullet or shrapnel injury. However, some biliary foreign bodies originate from ingested materials, such as teeth and bones. Of these, reports of fish bones are extremely rare, with only 16 reported cases [5, 6, 7, 8, 9, 10, 11, 12, 13, 14].

In our hospital, we have experienced several cases of cholangitis caused by fish bones in patients with a history of hepaticojejunostomy. In this study, we retrospectively reviewed all patients with fish bones identified as a biliary foreign body from the endoscopy database of Teine Keijinkai Hospital, describing the patients’ characteristics, treatment, and clinical courses.

Accordingly, we conducted this study to clarify the clinical characteristics, imaging features, and endoscopic treatment outcomes of fish bone migration into the bile duct after hepaticojejunostomy.

## 2 Methods

### 2.1 Patients

We enrolled patients who underwent endoscopic extraction of fish bones from the endoscopy database of the Teine Keijinkai Hospital between June 2016 and November 2023. The following data were collected from the electronic medical records: age, sex, prior history of biliary surgery, reason for performing endoscopy, imaging findings before treatment, time between biliary surgery and endoscopy, details of the endoscopic procedure, adverse events (AEs) after treatment based on the ASGE definition [15], and clinical course.

### 2.2 Treatment Strategy and Device Selection

It is essential to carefully review pre‐procedural CT images and intraprocedural fluoroscopic findings to confirm the number and precise locations of migrated fish bones, as multiple fish bones may migrate into the bile duct.

In patients who have undergone hepaticojejunostomy, reaching the anastomotic site using a conventional side‐viewing duodenoscope is often difficult. Therefore, a single‐balloon enteroscope with an overtube was used to reach the anastomosis.

When a fish bone was directly visible endoscopically at the hepaticojejunostomy site, forceps extraction was selected as the first‐line approach. Standard biopsy forceps were frequently used because of their sufficient grasping ability for elongated foreign bodies. In such cases, the forceps were carefully adjusted so that the long axis of the fish bone was aligned with the bile duct axis, thereby reducing the risk of bile duct injury during extraction.

When the fish bone was not visible at the anastomotic site or when biliary stones had formed around the fish bone as a nidus, basket extraction was selected as the first‐line approach. Previously, wire‐guided 8‐wire Dormia‐type baskets were mainly used. More recently, spiral‐type baskets, which are increasingly used in bile duct stone extraction, have also been employed, with rotation applied as needed to facilitate efficient retrieval of fish bones. During basket use, the deployment direction was also adjusted to align as closely as possible with the bile duct axis to enable safe extraction. In patients with an acute angle at the biliary branch, partial basket opening under guidewire guidance and gentle sliding along the duct facilitated appropriate advancement.

If resistance was encountered during extraction, there was a risk of bile duct injury due to the sharp edges of the fish bone. In such situations, the angle and position of the device were readjusted before further attempts. If resistance was encountered at the anastomotic site, balloon dilation was considered.

When removal using a single device was difficult, switching between multiple devices, including retrieval balloons selected according to the bile duct diameter, enabled successful extraction in most cases.

Because fish bones may fracture into multiple fragments during retrieval, the bile duct was swept several times to confirm the absence of residual fragments.

After extraction from the bile duct, the fish bones were retrieved extracorporeally through the scope when feasible. If through‐the‐scope retrieval was difficult, the fish bones were carefully withdrawn into the overtube under direct endoscopic visualization, avoiding mucosal injury.

Complete removal was defined as the absence of apparent residual fish bones on post‐procedural endoscopic images and cholangiographic findings.

Because most patients had undergone surgery for malignant disease, post‐procedural imaging follow‐up was mainly performed using CT in conjunction with routine surveillance for tumor recurrence or metastasis. Particular attention was paid to new linear high‐density structures in the bile duct suggestive of recurrent fish bone migration.

## 3 Results

### 3.1 Patient Characteristics

A review of our database between June 2016 and November 2023 revealed 9078 cases of ERCP procedures (8335 and 743 cases with normal and surgically altered anatomy, respectively), of which fish bones were extracted from 11 patients (1.5% of patients with surgically altered anatomy) (Table 1). All patients had a history of pancreaticobiliary neoplasms; overall, 10 underwent subtotal stomach‐preserving pancreaticoduodenectomies (SSPPD) with Child's reconstruction and one underwent extrahepatic bile duct resection with choledochojejunostomy. The median time between prior surgery and endoscopic treatment was 84 months (range 12–124). The most common reason for performing endoscopy was the detection of biliary stones or foreign bodies in the imaging diagnosis (54.5%), followed by the onset of cholangitis (27.3%), and elevated biliary enzymes (18.2%).

**TABLE 1:** Patient characteristics.

Number of patients, n	11	
Sex, male/female, n	9/2	
Median Age (range), years	76	(61‐83)
Primary disease		
Bile duct cancer, n	9	
Pancreatic cancer, n	2	
Adenoma of the ampulla of Vater, n	1	
Pervious surgical procedure		
Subtotal stomach‐preserving pancreaticoduodenectomy, n	10	
Extra hepatic bile duct resection, n	1	
Median time to diagnosis from operation (range), months	84	(12‐124)
Reason for performing endoscopy		
Foreign body or biliary stone on imaging examination, n	6	
Cholangitis, n	3	
Elevation of biliary enzymes, n	2	

**FIGURE 1:**
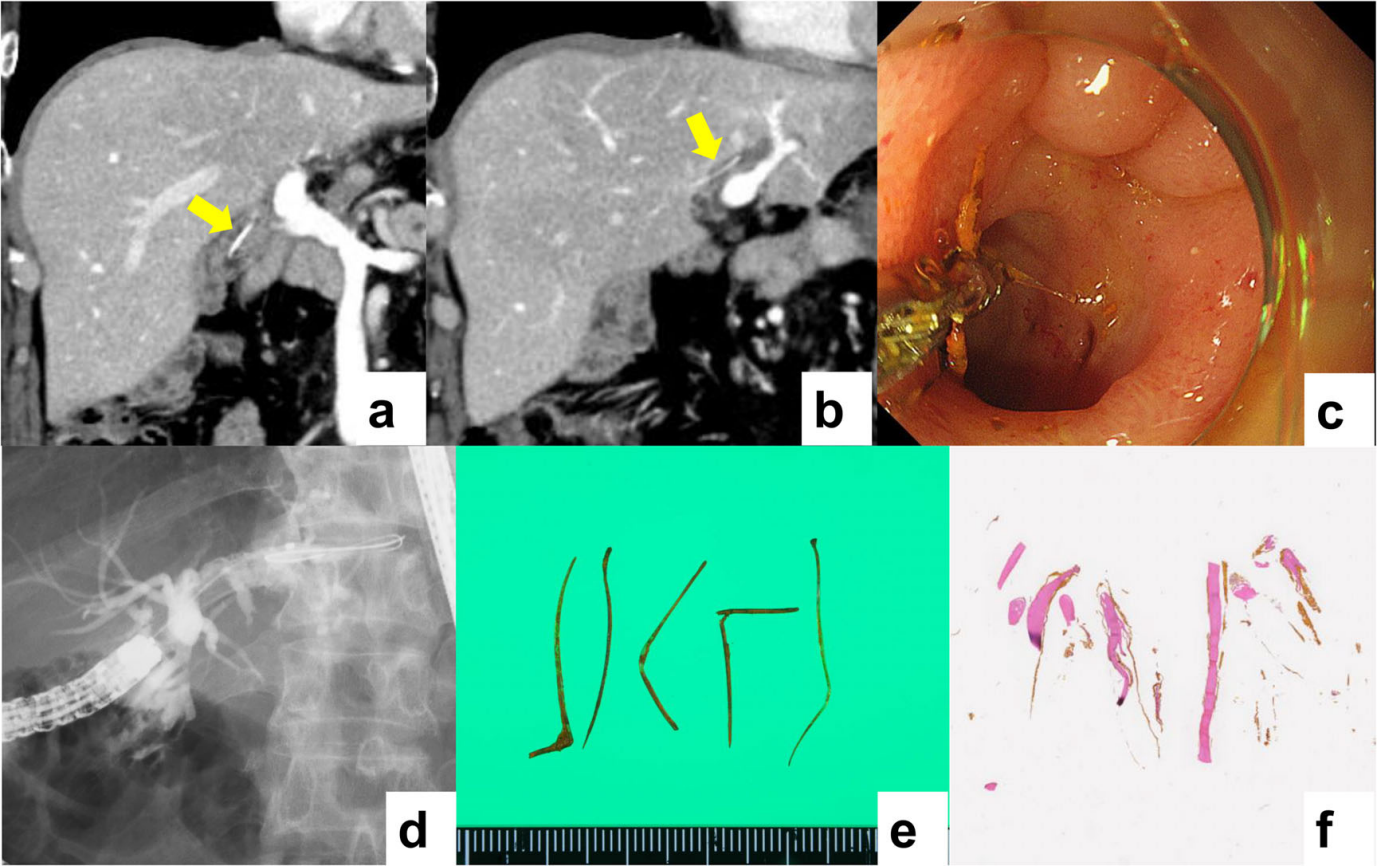
The patient was a 62‐year‐old man with a history of pancreaticoduodenectomy with reconstruction for bile duct cancer 7 years prior to requiring extraction of a fish bone. Highly dense linear materials were detected in the bile duct on enhanced computed tomography (a, b). All fish bones were successfully extracted using forceps via single‐balloon enteroscopy, without any adverse events (c, d, Video). Biliary stones were also removed by sweeping with a balloon catheter (Video). Five fish bones were removed from the patients (e), pathologically examined by Hematoxylin‐eosin staining, and found to be elongated, unstructured material with bile deposits, consistent with fish bones (f).

Regarding imaging examinations before the endoscopic procedure, computed tomography (CT) was performed in all patients. A retrospective review of CT imaging data revealed that foreign bodies could be visualized as high‐density dots or linear substances in the bile duct (Figure 1a,b). Differentiation of migrated fish bones from surgical clips after choledochojejunostomy or calcified debris was possible because fish bones showed intraductal positioning within the bile duct, a linear or slightly curved shape, and an absence of metal artifacts typically seen with surgical clips. Magnetic resonance imaging (MRI) and abdominal ultrasound (US) were performed in two and four patients, respectively; however, only a linear hyperechoic substance in the bile duct was detected in patients who underwent US.

### 3.2 Endoscopic Procedure

The details of the endoscopic procedures are summarized in Table 2. All procedures were performed using single‐balloon enteroscopy‐assisted ERCP in patients with surgically altered anatomies. Using a combination of forceps, basket catheters, and balloon catheters (Figure 1 c,d), all fish bones and 7 coexisting biliary stones were successfully removed. The median duration for fish bone removal (defined as the duration from endoscope insertion to withdrawal) was 40.5 (range 9–54) minutes. Overall, 3 cases of mild cholangitis were observed, all of which resolved with the administration of antibiotics. Of the 11 patients, histological interpretation was performed on 7 extracted samples. Histopathological examination with hematoxylin and eosin staining showed a bony matrix composed of collagen fibers, consistent with bone tissue. No cellular components suggestive of synthetic foreign bodies were observed (Figure 1 e,f). One patient had one recurrence, and another had two recurrences of biliary migration of the fish bone; however, all recurrent cases were successfully treated using single‐balloon enteroscopy.

**TABLE 2:** Details of endoscopic removal procedures.

Scope used (%), n		
SIF‐H290S	10	(90.1)
SIF‐Q260	1	(9.9)
Devices for removal (%), n		
Forceps alone	3	(27.3)
Basket alone	2	(18.2)
Balloon alone	1	(9.1)
Forceps + basket + balloon	1	(9.1)
Forceps + basket	1	(9.1)
Forceps + balloon	1	(9.1)
Basket + balloon	1	(9.1)
Median procedure time (range), min	40.5	(9‐54)
Median number of fish bones (range), n	2	(1‐13)
Coexistence of bile duct stones (%), n	7	(63.6)
Complete removal (%), n	11	(100)
Adverse events (%), n	3	(27.2)
Number of recurrence (%), n	2	(18.2)

## 4 Discussion

In the present study, we showed that migration of fish bones into the bile duct in patients who underwent biliary surgery is a rare but clinically significant complication.

In normal anatomy, unless endoscopic sphincterotomy for extraction of biliary stones or large‐bore stent placement for biliary stricture is performed, the migration of dietary contents into the bile duct is rare due to the action of the Oddi sphincter. In contrast, in cases with surgical anastomosis between the digestive tract and bile duct, dietary contents may easily enter the bile duct due to the sufficient diameter of the anastomosis for migration. However, biliary reconstruction surgery is performed by creating a jejunal afferent limb, and the migration of dietary contents is considered a rare phenomenon. Overall, there have been 10 prior studies investigating fish bone migration to the bile duct, including 16 case reports of fish bone migration to the bile duct, and 12 in patients with choledochojejunostomy (75.0%, Table 3). Considering the limited population of patients who undergo choledochojejunostomy, this procedure should be considered one of the strongest factors for the migration of fish bones [5, 6, 7, 8, 9, 10, 11, 12, 13, 14].

**TABLE 3:** Summary of prior case reports of fish bone migration into the bile duct after bile duct jejunal anastomosis.

No	Ref.no	Author	Year	Country	Age	Sex	Reason for treatment	Detection in imaging	Primary disease	Prior surgery	Time to diagnosis from surgery (month)	Treatment	Removal	Adverse event	Length of fish bone (mm)	Bile duct stone	Recurrence
1	5	Natusui	2022	Japan	66	M	cholangitis	CT	AC	PD	48	percutaneous	success	none	30	exist	none
2	6	Suzuki	2022	Japan	73	M	FB	CT	IPMN	PD	180	endoscopy	success	none	18	exist	none
3	7	Ishikawa	2021	Japan	67	M	FB	CT	PC	PD	36	operation	success	none	30	exist	none
4	8	Hirata	2021	Japan	70	M	cholangitis	CT	GC	DG	NA	endoscopy	success	none	20	none	none
5	9	Kunovsky	2021	Czech	79	F	FB	US	NA	NA	NA	endoscopy	success	none	30	none	none
6	10	Wu	2020	China	62	M	recurrence	NA	AC	PD	36	surgery	success	none	26	exist	none
7	11	Akahane	2019	Japan	50s	M	FB	CT	BDC	PD	12	SR	NA	NA	30	none	none
8	11	Akahane	2019	Japan	60s	F	FB	CT	DC	PD	18	SR	NA	NA	NA	none	none
9	11	Akahane	2019	Japan	70s	M	FB	CT	BDC	PD	16	SR	NA	NA	NA	none	none
10	11	Akahane	2019	Japan	70s	M	FB	CT	BDC	PD	9	SR	NA	NA	NA	none	unclear
11	11	Akahane	2019	Japan	60s	F	FB	CT	PC	PD	36	SR	NA	NA	NA	none	none
12	11	Akahane	2019	Japan	60s	M	FB	CT	PC	PD	21	SR	NA	NA	NA	exist	none
13	12	Koga	2018	Japan	71	M	cholangitis	CT	BDC	PD	12	endoscopy	success	none	unclear	none	none
14	13	Sakakida	2018	Japan	78	F	cholangitis	CT	DC	PD	108	endoscopy	success	none	20	exist	none
15	14	Kim	2004	Korea	75	F	cholangitis	CT	NA	NA	NA	percutaneous	success	none	15	exist	none
16	14	Kim	2004	Korea	67	M	cholangitis	PTC	NA	NA	NA	percutaneous	success	none	15	exist	none

FB, foreign body on image; CT, computed tomography; US, abdominal ultra sound; NA, not available; PTC, percutaneous cholangiography; AC, ampullary cancer; IPMN, intraductal papillary mucinous neoplasm; PC, pancreatic cancer; GC, gastric cancer; BDC, bile duct cancer; DC, duodenal cancer; PD, pancreaticoduodenectomy; DG, distal gastrectomy; SR, spontaneously removed.

One prior case series revealed that the majority of fish bones that migrated to the bile duct spontaneously disappeared during observation [11]. Of the 11 patients included in the present study, 8 were asymptomatic and could have been managed without aggressive treatment. However, foreign bodies in the bile duct are associated with stone formation in 69.8% of cases, and this rate is particularly high when the foreign bodies are derived from dietary contents [16]. The removal of biliary stones using balloon‐assisted enteroscopy, rather than percutaneous or surgical procedures, is currently performed safely and effectively in the majority of patients who undergo biliary surgery with reconstruction of the jejunal limbs [17]. All 11 patients successfully underwent removal of the fish bone without serious adverse events using single‐balloon enteroscopy. Thus, immediate removal of fish bones at the time of detection could be considered a treatment option.

In a retrospective review of CT scans prior to treatment, fish bones were detected as tiny or linear hyperdense materials in the bile duct in all patients (Figure 1 a,b). Biliary surgery with reconstruction is usually performed to cure pancreatic or biliary neoplasms. The majority of patients undergo planned imaging diagnosis as surveillance for recurrence and unexpected adverse events, and CT is the most common imaging modality. In such cases, careful review of the CT images would detect the migration of the fish bone into the bile duct before the appearance of clinical signs of biliary congestion. Furthermore, it is possible to determine the bile duct branches that have migrated using CT. Imaging before the endoscopic procedure is helpful to ensure an efficient and safe treatment (Figure 1a,b).

Previous reports describing pathological evaluation of migrated fish bones have demonstrated characteristic findings of bone tissue, including a bony matrix composed of collagen fibers on hematoxylin and eosin staining, and calcium‐phosphate–dominant composition on component analysis [8, 13, 16]. The histopathological features in our cases were consistent with these findings, supporting the diagnosis of migrated fish bones rather than other foreign materials.

This was a single‐center, retrospective case series study. From our database, we included 11 patients treated with an endoscopic procedure for migrating fish bones. However, not all patients who underwent biliary surgery were routinely followed with CT, and asymptomatic fish bone migration without biliary congestion may therefore have been overlooked. No fish bone migration was detected in patients without choledochjejunostomy who underwent ERCP; however, the endoscopist may have failed to detect the presence of fish bones in the core of the stones removed during the procedure. As such, the actual frequency of fish bone migration into the biliary tract remains unknown. This limitation will only be addressed in a prospective observational study.

In conclusion, this case series shows that the migration of fish bones into the bile duct is not uncommon in patients who undergo biliary surgery with reconstruction using the afferent limb. Further, our experience suggests that most migrated fish bones can be detected using CT and safely extracted using single‐balloon enteroscopy.

## Author Contributions

Kenta Yoshida, Tsuyoshi Hayashi, and Akio Katanuma conceived and designed the study, and drafted the initial manuscript. Kazuki Hama, Ryo Ando, Haruka Toyonaga, Tatsuya Ishii, Toshifumi Kin, Masayo Motoya, and Kuniyuki Takahashi contributed to case registration.Yuko Omori and Satoshi Ota performed the pathological diagnosis and histological evaluation. All authors contributed to manuscript preparation and have read and approved the final version.

## Funding Information

The authors received no specific funding for this work.

## Ethics Statement

This single‐center, retrospective, case series study was approved by the Institutional Review Board of Teine Keijinkai Hospital in Sapporo City, Hokkaido, Japan (approval number: 2‐023402‐00; registration date: 25/01/2024). Informed consent for participation was obtained using the opt‐out method listed on the hospital website.

## Conflicts of Interest

All authors declare no conflicts of interest. The study was not registered in a clinical trial registry (N/A). Animal studies were not applicable (N/A).

## Supporting information




**Video S1**: Endoscopic removal of a migrated fish bone from the bile duct using single‐balloon enteroscopy.
